# 3-(4-Bromo­phen­yl)-4-(4-hy­droxy­anilino)furan-2(5*H*)-one

**DOI:** 10.1107/S1600536811031849

**Published:** 2011-08-11

**Authors:** Wanxi Peng, Lansheng Wang, Fengjuan Wu, Qiu Xu

**Affiliations:** a School of Materials Science and Engineering, Central South University of Forestry and Technology, Changsha 410004, People’s Republic of China

## Abstract

In the title compound, C_16_H_12_BrNO_3_, the butyrolactone core adopts the furan-2(5*H*)-one structure and forms dihedral angles of 44.80 (17) and 65.73 (18)° with the bromo­benzene and phenol rings, respectively. In the crystal, N—H⋯O and O—H⋯O hydrogen bonds link the mol­ecules, generating *R*_4_^3^(26) loops The edge-fused rings extend to form a chain running along the *b*-axis direction and C—H⋯π contacts help to consolidate the packing.

## Related literature

For biological background to furan-2(5*H*)-one derivatives, see: Bailly *et al.* (2008 ▶); Weber *et al.* (2005 ▶); Xiao *et al.* (2011*a* ▶,*b* ▶). For related structures, see: Xiao *et al.* (2010 ▶, 2011*c* ▶).
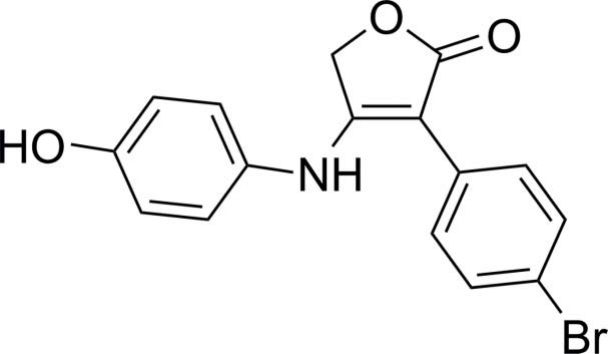


## Experimental

### 

#### Crystal data

C_16_H_12_BrNO_3_
*M* *_r_* = 346.18
Monoclinic, 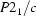

*a* = 11.2418 (10) Å
*b* = 8.0545 (7) Å
*c* = 16.1138 (13) Å
β = 100.244 (4)°
*V* = 1435.8 (2) Å^3^
*Z* = 4
Mo *K*α radiation
μ = 2.87 mm^−1^
*T* = 296 K
0.20 × 0.20 × 0.10 mm

#### Data collection

Bruker APEX CCD diffractometer
Absorption correction: multi-scan (*SADABS*; Sheldrick, 1996 ▶) *T*_min_ = 0.597, *T*_max_ = 0.762
7599 measured reflections
2725 independent reflections
1650 reflections with *I* > 2σ(*I*)
*R*_int_ = 0.035

#### Refinement

*R*[*F* ^2^ > 2σ(*F* ^2^)] = 0.041
*wR*(*F* ^2^) = 0.108
*S* = 1.01
2725 reflections
195 parameters
H atoms treated by a mixture of independent and constrained refinement
Δρ_max_ = 0.27 e Å^−3^
Δρ_min_ = −0.59 e Å^−3^

### 

Data collection: *SMART* (Bruker, 2000) ▶; cell refinement: *SAINT* (Bruker, 2000) ▶; data reduction: *SAINT* ▶; program(s) used to solve structure: *SHELXS97* (Sheldrick, 2008 ▶); program(s) used to refine structure: *SHELXL97* (Sheldrick, 2008 ▶); molecular graphics: *SHELXTL* (Sheldrick, 2008 ▶); software used to prepare material for publication: *SHELXTL*.

## Supplementary Material

Crystal structure: contains datablock(s) global, I. DOI: 10.1107/S1600536811031849/hb6341sup1.cif

Structure factors: contains datablock(s) I. DOI: 10.1107/S1600536811031849/hb6341Isup2.hkl

Supplementary material file. DOI: 10.1107/S1600536811031849/hb6341Isup3.cml

Additional supplementary materials:  crystallographic information; 3D view; checkCIF report

## Figures and Tables

**Table 1:** Hydrogen-bond geometry (Å, °) *Cg*1 is the centroid of the C1–C6 ring.

*D*—H⋯*A*	*D*—H	H⋯*A*	*D*⋯*A*	*D*—H⋯*A*
N1—H1⋯O1^i^	0.76 (4)	2.56 (4)	3.207 (4)	144 (4)
O3—H3*A*⋯O1^ii^	0.82	1.90	2.700 (4)	165
C12—H12⋯*Cg*1^i^	0.93	2.86	3.723 (4)	155

Symmetry codes: (i) 
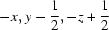
; (ii) 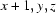.

**Fig. 1.**
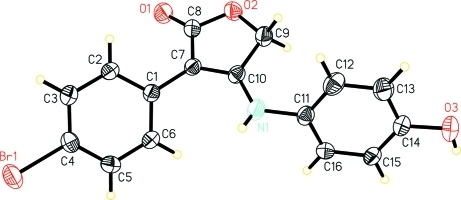
Molecular structure of the title compound, showing displacement ellipsoids drawn at the 30% probability level.

**Fig. 2.**
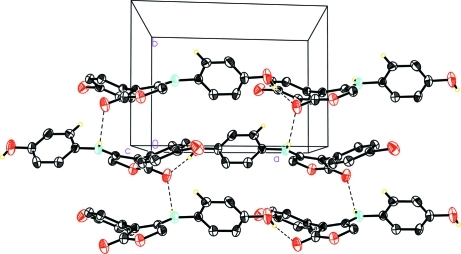
Motifs of R43(26) are formed through intermolecular N—H···O and O—H···O hydrogen bonds. For the sake of clarity, the H atoms not involved in the hydrogen bonds have been omitted.

**Fig. 3.**
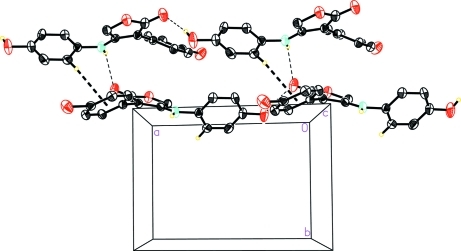
Packing diagram of compound (I) viewing along c axis, Solid dashed lines indicate C—H···π contacts. For the sake of clarity, the H atoms not involved in the hydrogen bonds have been omitted.

## supplementary crystallographic information

### Comment

Many compounds with γ-butyrolactone-core (furanone) show diverse biological activities such as antitumor and anti-inflammatory activity (Bailly *et al.*, 2008; Weber *et al.*, 2005). Recently, Xiao and his co-workers reported that 4-alkylamino or 4-arylamino derivatives of 3-arylfuran-2(5*H*)-one are potent inhibitors against tyrosyl-tRNA synthetase (TyrRS) (Xiao *et al.*, 2011*a* and 2011*b*), one of the aminoacyl-tRNA synthetases (aaRSs). Herein, we report the crystal structure of the title compound (I) (Fig. 1), an 3-aryl-4-arylaminofuran-2(5*H*)-one.

The bond C7—C10 (1.364 (4) Å) was assigned as a double bond, and the title compound was therefore identified as a furan-2(5*H*)-one (Xiao *et al.*, 2010; Xiao *et al.*, 2011*c*). C10—N1 (1.332 (4) Å) bond has shorter bond distance than the standard C—N single bond (1.48 Å), but longer than C—N double bond (1.28 Å). This clearly indicated that a *p* orbital of N1 is conjugated with the π molecular orbital of C7—C10 double bond. However, the bond distance of C11—N1 is 1.437 (4) Å, much longer than that of C10—N1. This may be caused by the large dihedral angle [65.76 (28) °] between the amino group (C10, C11, N1 and H1) and the 4-hydroxybenzene ring, which significantly disrupted the conjugation between N1 and its attached benzene ring.

In the crystal, four molecules of I are connected by intermolecular N1—H1···O1 and O3—H3A···O1 interactions to generate a ring motif described by a graph-set motif of *R*_4_^3^(26) (Fig. 2). The edge-fused rings extend to form a sheet running along the *b* axis. The resulted sheet is further stablized by C—H···π contacts (Fig. 3).

### Experimental

3-(4-bromophenyl)-4-hydroxyfuran-2(5*H*)-one (255 mg, 1 mmol) was prepared according to the procedure described by Xiao (Xiao *et al.*, 2011*a*), which was added into a mixture of 4-hydroxyaniline (130 mg, 1.2 mmol) and *p*-toluene sulphonic acid (6.8 mg, 0.04 mmol). The resulted mixture was heated to 370 K for 10 min. Nine ml of toluene was then added and refluxed for 6 h. After toluene was removed under reduced pressure, the residue was purified by column chromatography on silica gel, eluting with EtOAc/petroleum ether (*v*/*v* = 2/1), which furnished colorless blocks of I by slow evaporation at room temperature.

### Refinement

The H atom bonded to N1 was located in difference Fourier maps, and all other H atoms were placed in geometrically idealized positions and constrained to ride on their parent atoms with C—H = 0.93 Å for aromatic H atoms, 0.97 Å for CH_2_ type H atoms and O—H = 0.82 Å hydroxyl group, respectively. *U*_iso_(H) values were set at 1.2 times *U*_eq_(C) for all H atoms, and at 1.5 times *U*_eq_(O) for hydrogxyl group.

### Crystal data

**Table:**

C_16_H_12_BrNO_3_	*F*(000) = 696
*M**_r_* = 346.18	*D*_x_ = 1.601 Mg m^−^^3^
Monoclinic, *P*2_1_/*c*	Mo *K*α radiation, λ = 0.71073 Å
Hall symbol: -P 2ybc	Cell parameters from 1582 reflections
*a* = 11.2418 (10) Å	θ = 2.1–25.1°
*b* = 8.0545 (7) Å	µ = 2.87 mm^−^^1^
*c* = 16.1138 (13) Å	*T* = 296 K
β = 100.244 (4)°	Block, colorless
*V* = 1435.8 (2) Å^3^	0.20 × 0.20 × 0.10 mm
*Z* = 4	

### Data collection

**Table:**

Bruker APEX CCD diffractometer	2725 independent reflections
Radiation source: fine-focus sealed tube	1650 reflections with *I* > 2σ(*I*)
graphite	*R*_int_ = 0.035
φ and ω scans	θ_max_ = 25.8°, θ_min_ = 1.8°
Absorption correction: multi-scan (*SADABS*; Sheldrick, 1996)	*h* = −13→10
*T*_min_ = 0.597, *T*_max_ = 0.762	*k* = −9→9
7599 measured reflections	*l* = −18→19

### Refinement

**Table:**

Refinement on *F*^2^	Primary atom site location: structure-invariant direct methods
Least-squares matrix: full	Secondary atom site location: difference Fourier map
*R*[*F*^2^ > 2σ(*F*^2^)] = 0.041	Hydrogen site location: inferred from neighbouring sites
*wR*(*F*^2^) = 0.108	H atoms treated by a mixture of independent and constrained refinement
*S* = 1.01	*w* = 1/[σ^2^(*F*_o_^2^) + (0.0519*P*)^2^ + 0.1637*P*] where *P* = (*F*_o_^2^ + 2*F*_c_^2^)/3
2725 reflections	(Δ/σ)_max_ < 0.001
195 parameters	Δρ_max_ = 0.27 e Å^−^^3^
0 restraints	Δρ_min_ = −0.59 e Å^−^^3^

### Special details

**Table:**

Geometry. All e.s.d.'s (except the e.s.d. in the dihedral angle between two l.s. planes) are estimated using the full covariance matrix. The cell e.s.d.'s are taken into account individually in the estimation of e.s.d.'s in distances, angles and torsion angles; correlations between e.s.d.'s in cell parameters are only used when they are defined by crystal symmetry. An approximate (isotropic) treatment of cell e.s.d.'s is used for estimating e.s.d.'s involving l.s. planes.
Refinement. Refinement of *F*^2^ against ALL reflections. The weighted *R*-factor *wR* and goodness of fit *S* are based on *F*^2^, conventional *R*-factors *R* are based on *F*, with *F* set to zero for negative *F*^2^. The threshold expression of *F*^2^ > σ(*F*^2^) is used only for calculating *R*-factors(gt) *etc*. and is not relevant to the choice of reflections for refinement. *R*-factors based on *F*^2^ are statistically about twice as large as those based on *F*, and *R*- factors based on ALL data will be even larger.

### Fractional atomic coordinates and isotropic or equivalent isotropic displacement parameters (Å^2^)

**Table:**

	*x*	*y*	*z*	*U*_iso_*/*U*_eq_	
Br1	−0.33348 (4)	0.01420 (6)	−0.05871 (2)	0.0870 (2)	
C1	−0.0793 (3)	0.0631 (4)	0.2028 (2)	0.0449 (8)	
C2	−0.1939 (3)	−0.0066 (4)	0.1953 (2)	0.0506 (8)	
H2	−0.2201	−0.0450	0.2435	0.061*	
C3	−0.2695 (3)	−0.0201 (4)	0.1185 (2)	0.0544 (9)	
H3	−0.3466	−0.0647	0.1148	0.065*	
C4	−0.2294 (3)	0.0333 (4)	0.0473 (2)	0.0512 (9)	
C5	−0.1167 (3)	0.1009 (4)	0.0516 (2)	0.0549 (9)	
H5	−0.0908	0.1361	0.0028	0.066*	
C6	−0.0417 (3)	0.1162 (4)	0.1293 (2)	0.0522 (8)	
H6	0.0347	0.1625	0.1324	0.063*	
C7	−0.0029 (3)	0.0832 (4)	0.28604 (19)	0.0452 (8)	
C8	−0.0498 (3)	0.1479 (4)	0.3561 (2)	0.0548 (9)	
C9	0.1485 (3)	0.0909 (4)	0.4049 (2)	0.0549 (9)	
H9A	0.1731	−0.0102	0.4360	0.066*	
H9B	0.2132	0.1718	0.4172	0.066*	
C10	0.1178 (3)	0.0571 (4)	0.31223 (19)	0.0447 (8)	
C11	0.3288 (3)	0.0027 (4)	0.3037 (2)	0.0485 (8)	
C12	0.3717 (3)	−0.1054 (4)	0.3681 (2)	0.0632 (10)	
H12	0.3191	−0.1784	0.3879	0.076*	
C13	0.4933 (3)	−0.1054 (5)	0.4032 (2)	0.0690 (10)	
H13	0.5224	−0.1783	0.4469	0.083*	
C14	0.5709 (3)	0.0011 (5)	0.3740 (2)	0.0603 (10)	
C15	0.5287 (3)	0.1075 (5)	0.3088 (2)	0.0575 (9)	
H15	0.5817	0.1787	0.2881	0.069*	
C16	0.4072 (3)	0.1084 (4)	0.27397 (19)	0.0517 (8)	
H16	0.3784	0.1810	0.2301	0.062*	
N1	0.2020 (3)	0.0087 (4)	0.2687 (2)	0.0594 (8)	
O1	−0.1497 (2)	0.2035 (4)	0.36051 (14)	0.0755 (8)	
O2	0.03891 (19)	0.1547 (3)	0.42643 (13)	0.0621 (6)	
O3	0.6898 (2)	−0.0049 (4)	0.4118 (2)	0.0954 (10)	
H3A	0.7279	0.0661	0.3911	0.143*	
H1	0.187 (3)	−0.027 (4)	0.224 (2)	0.062 (13)*	

### Atomic displacement parameters (Å^2^)

**Table:**

	*U*^11^	*U*^22^	*U*^33^	*U*^12^	*U*^13^	*U*^23^
Br1	0.0902 (4)	0.1047 (4)	0.0567 (3)	−0.0269 (2)	−0.0122 (2)	0.0012 (2)
C1	0.0419 (18)	0.0458 (18)	0.0469 (18)	−0.0003 (14)	0.0078 (14)	−0.0035 (15)
C2	0.046 (2)	0.057 (2)	0.0502 (19)	−0.0069 (16)	0.0130 (15)	0.0000 (16)
C3	0.047 (2)	0.057 (2)	0.058 (2)	−0.0081 (16)	0.0074 (16)	−0.0011 (17)
C4	0.057 (2)	0.043 (2)	0.051 (2)	0.0020 (16)	0.0011 (16)	−0.0051 (15)
C5	0.061 (2)	0.060 (2)	0.0455 (19)	−0.0004 (18)	0.0133 (16)	0.0042 (16)
C6	0.0436 (18)	0.060 (2)	0.055 (2)	−0.0065 (16)	0.0131 (15)	0.0012 (17)
C7	0.0409 (18)	0.0498 (19)	0.0460 (18)	−0.0022 (15)	0.0109 (14)	−0.0043 (15)
C8	0.045 (2)	0.070 (2)	0.049 (2)	−0.0012 (18)	0.0066 (16)	−0.0012 (17)
C9	0.046 (2)	0.060 (2)	0.057 (2)	0.0035 (17)	0.0053 (16)	−0.0054 (18)
C10	0.0414 (19)	0.0432 (18)	0.0503 (19)	−0.0064 (14)	0.0100 (15)	−0.0048 (14)
C11	0.0401 (19)	0.054 (2)	0.0519 (19)	0.0012 (16)	0.0089 (15)	−0.0112 (17)
C12	0.055 (2)	0.050 (2)	0.088 (3)	−0.0011 (17)	0.021 (2)	0.0051 (19)
C13	0.060 (2)	0.070 (3)	0.077 (3)	0.019 (2)	0.013 (2)	0.016 (2)
C14	0.039 (2)	0.080 (3)	0.062 (2)	0.0043 (18)	0.0085 (17)	0.003 (2)
C15	0.0407 (19)	0.078 (3)	0.055 (2)	−0.0114 (17)	0.0119 (16)	0.0001 (19)
C16	0.050 (2)	0.066 (2)	0.0410 (18)	0.0016 (18)	0.0111 (15)	−0.0016 (16)
N1	0.0412 (17)	0.079 (2)	0.058 (2)	−0.0017 (14)	0.0086 (15)	−0.0229 (18)
O1	0.0518 (15)	0.115 (2)	0.0616 (16)	0.0171 (14)	0.0139 (12)	−0.0101 (14)
O2	0.0556 (14)	0.0847 (18)	0.0457 (13)	0.0084 (12)	0.0087 (11)	−0.0092 (12)
O3	0.0431 (16)	0.147 (3)	0.091 (2)	0.0103 (15)	−0.0021 (14)	0.0237 (18)

### Geometric parameters (Å, °)

**Table:**

Br1—C4	1.897 (3)	C9—H9A	0.9700
C1—C2	1.391 (4)	C9—H9B	0.9700
C1—C6	1.394 (4)	C10—N1	1.332 (4)
C1—C7	1.467 (4)	C11—C16	1.371 (4)
C2—C3	1.376 (5)	C11—C12	1.375 (5)
C2—H2	0.9300	C11—N1	1.437 (4)
C3—C4	1.373 (5)	C12—C13	1.384 (5)
C3—H3	0.9300	C12—H12	0.9300
C4—C5	1.369 (5)	C13—C14	1.366 (5)
C5—C6	1.384 (4)	C13—H13	0.9300
C5—H5	0.9300	C14—O3	1.368 (4)
C6—H6	0.9300	C14—C15	1.373 (5)
C7—C10	1.364 (4)	C15—C16	1.381 (4)
C7—C8	1.428 (4)	C15—H15	0.9300
C8—O1	1.222 (4)	C16—H16	0.9300
C8—O2	1.371 (3)	N1—H1	0.76 (4)
C9—O2	1.433 (4)	O3—H3A	0.8200
C9—C10	1.497 (4)		
			
C2—C1—C6	117.8 (3)	C10—C9—H9B	110.8
C2—C1—C7	120.4 (3)	H9A—C9—H9B	108.9
C6—C1—C7	121.8 (3)	N1—C10—C7	130.0 (3)
C3—C2—C1	121.6 (3)	N1—C10—C9	121.3 (3)
C3—C2—H2	119.2	C7—C10—C9	108.7 (3)
C1—C2—H2	119.2	C16—C11—C12	119.7 (3)
C4—C3—C2	119.0 (3)	C16—C11—N1	119.8 (3)
C4—C3—H3	120.5	C12—C11—N1	120.5 (3)
C2—C3—H3	120.5	C11—C12—C13	119.8 (3)
C5—C4—C3	121.4 (3)	C11—C12—H12	120.1
C5—C4—Br1	119.6 (3)	C13—C12—H12	120.1
C3—C4—Br1	119.0 (3)	C14—C13—C12	120.2 (3)
C4—C5—C6	119.3 (3)	C14—C13—H13	119.9
C4—C5—H5	120.3	C12—C13—H13	119.9
C6—C5—H5	120.3	C13—C14—O3	117.2 (3)
C5—C6—C1	120.9 (3)	C13—C14—C15	120.1 (3)
C5—C6—H6	119.6	O3—C14—C15	122.7 (3)
C1—C6—H6	119.6	C14—C15—C16	119.7 (3)
C10—C7—C8	107.4 (3)	C14—C15—H15	120.1
C10—C7—C1	130.9 (3)	C16—C15—H15	120.1
C8—C7—C1	121.6 (3)	C11—C16—C15	120.4 (3)
O1—C8—O2	118.6 (3)	C11—C16—H16	119.8
O1—C8—C7	130.7 (3)	C15—C16—H16	119.8
O2—C8—C7	110.6 (3)	C10—N1—C11	123.4 (3)
O2—C9—C10	104.5 (2)	C10—N1—H1	123 (3)
O2—C9—H9A	110.8	C11—N1—H1	113 (3)
C10—C9—H9A	110.8	C8—O2—C9	108.4 (2)
O2—C9—H9B	110.8	C14—O3—H3A	109.5
			
C6—C1—C2—C3	−1.3 (5)	C1—C7—C10—C9	−177.8 (3)
C7—C1—C2—C3	177.0 (3)	O2—C9—C10—N1	174.5 (3)
C1—C2—C3—C4	1.5 (5)	O2—C9—C10—C7	−6.0 (4)
C2—C3—C4—C5	−0.7 (5)	C16—C11—C12—C13	0.9 (5)
C2—C3—C4—Br1	179.8 (2)	N1—C11—C12—C13	−177.6 (3)
C3—C4—C5—C6	−0.2 (5)	C11—C12—C13—C14	−0.3 (6)
Br1—C4—C5—C6	179.3 (2)	C12—C13—C14—O3	179.7 (4)
C4—C5—C6—C1	0.3 (5)	C12—C13—C14—C15	−0.8 (6)
C2—C1—C6—C5	0.4 (5)	C13—C14—C15—C16	1.2 (5)
C7—C1—C6—C5	−177.9 (3)	O3—C14—C15—C16	−179.3 (3)
C2—C1—C7—C10	138.5 (4)	C12—C11—C16—C15	−0.5 (5)
C6—C1—C7—C10	−43.2 (5)	N1—C11—C16—C15	178.0 (3)
C2—C1—C7—C8	−45.2 (4)	C14—C15—C16—C11	−0.5 (5)
C6—C1—C7—C8	133.1 (3)	C7—C10—N1—C11	173.4 (3)
C10—C7—C8—O1	172.4 (4)	C9—C10—N1—C11	−7.2 (5)
C1—C7—C8—O1	−4.6 (6)	C16—C11—N1—C10	−113.5 (4)
C10—C7—C8—O2	−2.9 (4)	C12—C11—N1—C10	64.9 (5)
C1—C7—C8—O2	−180.0 (3)	O1—C8—O2—C9	−177.0 (3)
C8—C7—C10—N1	−175.1 (3)	C7—C8—O2—C9	−1.0 (4)
C1—C7—C10—N1	1.6 (6)	C10—C9—O2—C8	4.2 (3)
C8—C7—C10—C9	5.5 (4)		

### Hydrogen-bond geometry (Å, °)

**Table:**

Cg1 is the centroid of the C1–C6 ring.

**Table:**

*D*—H···*A*	*D*—H	H···*A*	*D*···*A*	*D*—H···*A*
N1—H1···O1^i^	0.76 (4)	2.56 (4)	3.207 (4)	144 (4)
O3—H3A···O1^ii^	0.82	1.90	2.700 (4)	165.
C12—H12···Cg1^i^	0.93	2.86	3.723 (4)	155

Symmetry codes: (i) −*x*, *y*−1/2, −*z*+1/2; (ii) *x*+1, *y*, *z*.
